# A Big World Inside Small-World Networks

**DOI:** 10.1371/journal.pone.0005686

**Published:** 2009-05-25

**Authors:** Zhihua Zhang, Jianzhi Zhang

**Affiliations:** Department of Ecology and Evolutionary Biology, University of Michigan, Ann Arbor, Michigan, United States of America; Georgia Institute of Technology, United States of America

## Abstract

Real networks, including biological networks, are known to have the small-world property, characterized by a small “diameter”, which is defined as the average minimal path length between all pairs of nodes in a network. Because random networks also have short diameters, one may predict that the diameter of a real network should be even shorter than its random expectation, because having shorter diameters potentially increases the network efficiency such as minimizing transition times between metabolic states in the context of metabolic networks. Contrary to this expectation, we here report that the observed diameter is greater than the random expectation in every real network examined, including biological, social, technological, and linguistic networks. Simulations show that a modest enlargement of the diameter beyond its expectation allows a substantial increase of the network modularity, which is present in all real networks examined. Hence, short diameters appear to be sacrificed for high modularities, suggesting a tradeoff between network efficiency and advantages offered by modularity (e.g., multi-functionality, robustness, and/or evolvability).

## Introduction

Network diameter (*D*) has long been of interest. The pioneering finding of Milgram [1] that two random individuals can connect to each other through on average 5–6 intermediate steps suggested that the human acquaintanceship network is a small world, prompting the popular phrase “six-degrees of separation”. There appear to be several different descriptions of the small-world property, but it is the small diameter that is referred to throughout this article. Biological networks such as metabolic networks and protein interaction networks also show the small-world property [2]–[4]. In the context of metabolic networks, the small-world architecture has been suggested to serve to minimize transition times between metabolic states [3]. However, subsequent theoretical work demonstrated that even random (irregular) networks, including the simplest one that is formed by connecting nodes entirely randomly (known as the Erdős-Rényi or ER network [5]), show small diameters, having 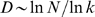, where *N* is the number of nodes in the network and *k* is the mean number of edges per node (i.e., mean degree) [6]. In ER networks, node degree follows a Poisson distribution. In real networks, however, node degree often approximates power-law distributions [7]. It has been shown that random power-law networks with exponents between 2 and 3 have 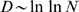, sometimes referred to as the ultra-small-world property [8]. Because even random networks have the small-world property, it is of no surprise that real networks also show this character. Nevertheless, an interesting question is whether the diameter of a real network is even shorter than its random expectation, because having short diameters can potentially increase the network efficiency of exchanging mass and/or information [9], [10], not only in biological networks (e.g., the metabolic network), but also in transportation, communication, and computer networks [11], [12]. Watts and Strogatz reported that the diameters of three real networks (power grid, film actors, and nematode neural network) are larger than those of ER random networks with the same numbers of nodes and mean degrees [6]. However, it is unclear (1) whether the difference in diameter is statistically significant, (2) whether a greater-than-expected diameter is generally true in all real networks, and (3) most importantly, whether their observation is simply caused by the use of an inappropriate null model (ER) for real networks. We here address these questions and report the unexpected finding that all real networks analyzed have greater-than-expected diameters and discern the cause of this phenomenon.

## Results and Discussion

### Real networks have greater-than-expected diameters

We compare the diameter of a real network with that of its randomly rewired network in which the connections between nodes are randomized while the degree of every node remains unchanged (see Methods). We find that all 13 real networks examined, including two linguistic, three technological, four social, and four biological networks, have diameters greater than their random expectations (Table 1). Frequency distributions of minimal path lengths show that the greater-than-expected diameters are not caused by the presence of a small number of extraordinarily long minimal paths in the networks, but due to the existence of many elongated minimal paths (Fig. S1). Overall, the diameters of real networks are 2.3–128.3% greater than their random expectations, with a median difference of 17.4% (Table 1). Consistent with our findings, Albert and Barabási briefly noted that many real networks have longer diameters than those computed under the power-law degree distribution [13], an observation that may be explained by the deviation of the actual degree distribution from the power-law distribution [14]. In our analysis, however, this explanation can be ruled out because we used the true degree distribution in estimating the expected diameter.

**Table 1:** The diameters and modularities of 13 real networks.

		Diameter					Modularity				
Networks	# of nodes	Observed	Expected	% difference	*Z*-score^14^	*P-*value	Observed	Expected	% difference	*Z*-score^14^	*P*-value
Characters in “Les Miserables”^1^	77	2.64	2.50	5.6	3.58	0.0003	0.56	0.29	93.4	30.12	<10^−4^
Words in “David Copperfield”^2^	112	2.54	2.48	2.3	1.81	0.0703	0.31	0.29	4.8	1.67	0.0949
Dolphins^3^	62	3.36	2.70	24.3	14.40	<10^−4^	0.53	0.37	40.8	11.59	<10^−4^
Political blogs^4^	1224	2.74	2.59	5.7	23.5	<10^−4^	0.43	0.14	206.9	189.27	<10^−4^
Co-authorship^5^	7610	7.03	5.42	29.6	64.70	<10^−4^	0.81	0.49	64.9	12.50	<10^−4^
Football^6^	115	2.51	2.23	12.5	54.30	<10^−4^	0.60	0.28	119.2	44.68	<10^−4^
Power^7^	4941	18.99	8.32	128.3	14.30	<10^−4^	0.93	0.73	28.5	105.10	<10^−4^
Airline^8^	810	3.06	2.61	17.4	3.53	0.0004	0.31	0.13	130.0	114.70	<10^−4^
Electronic circuits^9^	512	6.86	5.64	21.6	12.40	<10^−4^	0.81	0.63	28.6	35.96	<10^−4^
Protein-protein interaction^10^	1870	6.81	5.78	17.8	9.19	<10^−4^	0.81	0.72	13.2	18.23	<10^−4^
Neural^11^	297	2.46	2.35	4.5	3.38	0.0007	0.40	0.22	80.0	51.26	<10^−4^
Transcriptional regulatory^12^	3459	3.72	3.39	9.7	3.60	0.0003	0.60	0.47	29.5	58.29	<10^−4^
Metabolic^13^	563	8.78	6.54	34.3	18.67	<10^−4^	0.84	0.73	14.5	14.72	<10^−4^

1 The network of coappearances of characters in Victor Hugo's novel “Les Miserables”. Nodes represent characters and edges connect any pair of characters that appear in the same chapter.

2 The network of common adjective and noun adjacencies for the novel “David Copperfield” by Charles Dickens. Nodes represent the most commonly occurring adjectives and nouns in the book.

3 The network of frequent associations between 62 dolphins in a community living off Doubtful Sound, New Zealand.

4 The network of political blogs. Nodes represent blogs and edges are the links between blogs.

5 The network of scientists posting preprints on the high-energy theory archive at www.arxiv.org, 1995–1999. Nodes are authors and edges connect coauthors.

6 The network of American football games between Division IA colleges during regular season Fall 2000. Nodes are teams and edges connect teams that contest in a game.

7 The network of the Western States Power Grid of the United States. Nodes are power plants, stations and households, and edges are powerlines.

8 The network of scheduled air line connections in United States, 2005. Nodes are airports and edges are scheduled direct flights.

9 Electronic circuits. Nodes are electronic elements and edges are electronic connections.

10 The protein-protein interaction network of the budding yeast *S. cerevisiae*. Nodes are proteins and edges connect proteins that interact with each other.

11 The neural network for the worm *C. elegans*. Nodes are neurons and edges link neurons that connect.

12 The transcriptional regulatory network of the budding yeast *S. cerevisiae*. Nodes are genes and edges connect genes that regulate one another.

13 The metabolic network of the bacterium *E. coli*. Nodes are metabolites and edges connect metabolites that can be converted by a biochemical reaction.

14 *Z*-score, number of standard deviations by which the observation deviates from the expectation.

### Real networks have greater-than-expected clustering coefficients

Another commonly described feature of small-world networks is a high clustering coefficient (*C*) [6]. The clustering coefficient for a node is defined by the proportion of links between the nodes within its neighbourhood divided by the number of links that could possibly exist between them [6]. The clustering coefficient of a network is the mean clustering coefficient of all nodes in the network [6]. We found that in 12 of the 13 real networks (except the dolphin association network), *C* is greater than the expected value determined from its randomly rewired networks, and this difference is statistically significant in 7 cases (Table S1). However, there is no consistent relationship between *C* and *D* among the randomly rewired networks of each real network (Table S1), suggesting that the greater-than-expected diameters of real networks cannot be explained by the greater-than-expected clustering coefficients. Furthermore, across the 13 real networks, the correlation between the expected *C* and expected *D* from their randomly rewired networks appears negatively (Spearman's *R* = −0.57, *P* = 0.047), while the correlation between the *Z*-score for *C* and *Z*-score for *D* is not significant (Spearman's *R* = −0.13, *P* = 0.68).

Here *Z*-score refers to the number of standard deviations by which an observed *C* (or *D*) deviates from its chance expectation in a randomly rewired network.

### Network modularization enlarges the diameter

**Figure 1:**
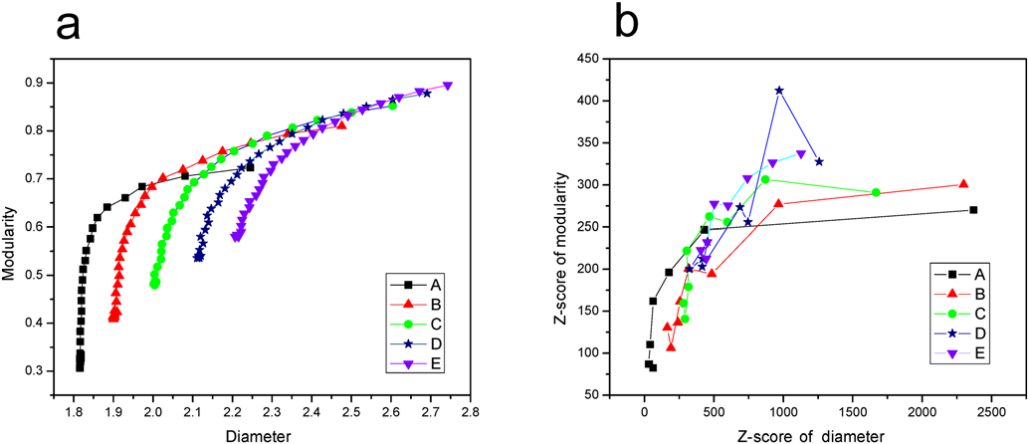
Correlation between network diameter and modularity in simulated networks when diameter and modularity are measured in (a) absolute values and (b) *Z*-scores. Each point represents a network and each line connects the networks of the same series. The number of modules is fixed at 4, 6, 8, 10 and 12 for series A, B, C, D, and E, respectively. Within each network series, the ratio (*R*) of the number of between-module edges to that of within-module edges changes from 30∶1 to 1∶30 so that modularity gradually increases. The same pattern is observed when we examine the relationships of mean diameter and mean modularity of 50 randomly rewired networks of a simulated network with preserved modules (see Fig. S3). In (b), 8 networks are shown for each series to allow clarity of the figure (*R* = 1∶30, 5∶26, 9∶22, 13∶18, 17∶14, 21∶10, 25∶6, and 29∶2, respectively). Z-score is the number of standard deviations by which an observed value deviates from its expected value. Here the expected value and the standard deviation are estimated by random network rewiring.

If shorter diameters are beneficial to at least some networks, why do all networks have longer diameters than expected by chance? We hypothesize that this phenomenon relates to the modularization in networks, which refers to the fact that networks can often be divided into sets of nodes (i.e., modules) such that links within modules are much denser than between modules. Modularization could lead to the enlargement of the network diameter because it increases the minimal path length between modules and because there are usually more pairs of nodes across modules than within modules in a highly modular network. To verify our hypothesis, we conduct computer simulations. In each set of simulations, we fix the numbers of nodes and edges in a network but adjust the connections to increase the modularity. Briefly, a random network is generated from *m* fully connected modules that are interlinked by one edge. At each step, a new node with *K* intra-module edges and *S* inter-module edges is randomly added to a module. These edges are attached to existing nodes via the preferential attachment model (see Methods). The degree distribution of the generated network was reported to approach power-law [15]. By adjusting parameters *K* and *S*, we can generate networks with desired modularity. We find that the network diameter increases as the modularity increases in these simulated networks (Fig. 1a). But the relationship between diameter and modularity is not linear; when the diameter is short, a small percentage increase in diameter allows a substantial percentage increase in modularity (Fig. 1a). A similar concave curve is observed when the increases in diameter and modularity are measured by *Z*-scores, rather than the absolute values (Fig. 1b). Using a similar simulation, we confirmed the relationship between modularity and diameter using networks with a fixed number of modules but different mean degrees (Fig. S2).

If modularization is truly the cause of the higher-than-expected diameters of real networks, all real networks should have modularities greater than expected from their randomly rewired networks. This is indeed the case [16] (see also Table 1). The percentage excess in modularity (compared to the random expectation) ranges from 4.8 to 206.9% for the 13 networks, with a median of 40.8%. This percentage excess exceeds that for diameter in 10 of the 13 networks, a nonrandom pattern that is consistent with the simulation result in Fig. 1 (*P* = 0.046, one-tail binomial test).

**Figure 2:**
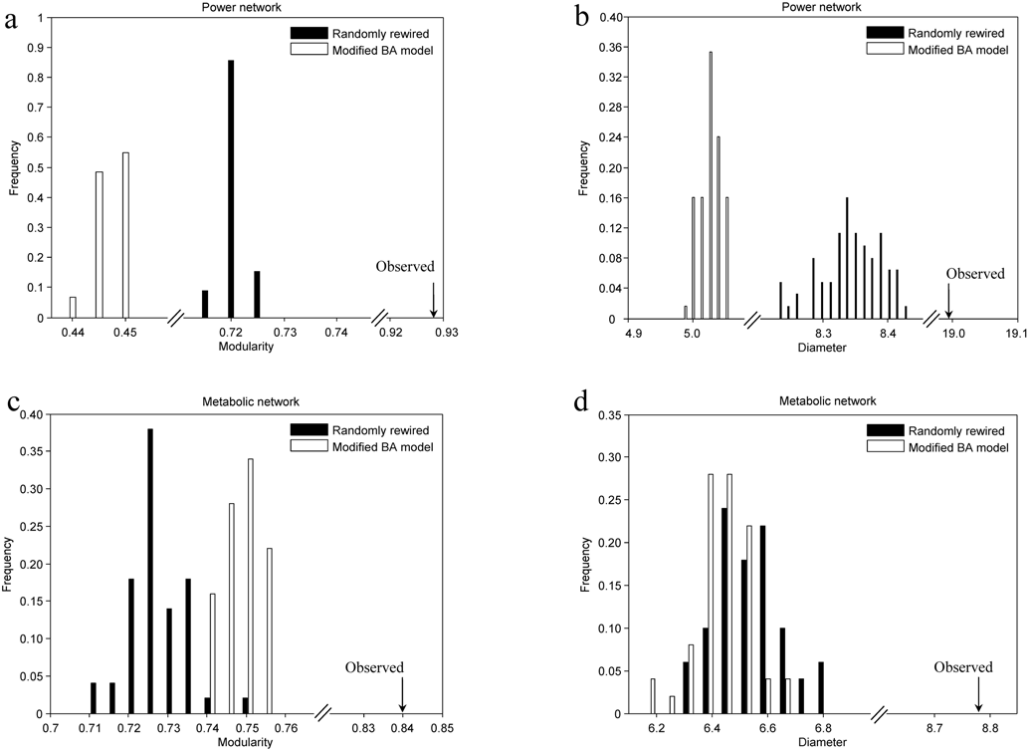
Observed and expected modularities and diameters of the power and metabolic networks. The top panels represent the power network and the bottom panels represent the metabolic network.

The observation that the modularity of a real network is greater than that of its randomly rewired networks does not prove that high modularity is a design principle of real networks, as high modularity may arise as a byproduct of other processes, such as the evolution by gene duplication process in the growth of some biological networks [17]. Here we investigate whether the preferential attachment model of Barabási and Albert (BA model), a widely used model for generating power-law networks with exponent >2 [7], [18], can explain the observed high modularity. Among the 13 real networks, the power and metabolic networks have exponents greater than 2 (2.75 and 2.40 respectively). We use a modified BA model to grow networks that have the same numbers of nodes and edges as the observed networks (see Methods). We then compare the modularity of the real, randomly rewired, and BA-model networks. In both power and metabolic networks, the observed modularity is significantly greater than the modularity of the BA-model networks and that of randomly rewired networks (Fig. 2a, 2c). Similar results are found for the diameter (Fig. 2b, 2d). Because other models for generating power-law networks are in principle similar to the BA model [13], [19]–[21], it is unlikely that the high modularity of the two real networks can be explained by these other models. Rather, the high modularity may have been directly favored in these networks [22]. Computer simulation shows that modular structures can arise when a network faces multiple alternating tasks [23]. On the one hand, high modularity allows a system to acquire and abandon functional units without causing pleiotropic effects, thus improving the evolvability of the system. On the other hand, numerical experiments also demonstrated that modularization provides robustness against random perturbations in network structure [24], presumably also due to the separation of different functions by modules. These benefits of modularity have been used practically such as in software design, where individual functions are assigned to distinct modules and the software is then assembled by connecting different modules [25], [26]. Diameter is apparently not as much of a concern as modularity in software design.

It is interesting to ask if modularization is the sole reason of the higher-than-expected diameters in real networks. We conducted a second type of random network rewiring, by conserving the modular structure of the network as well as the within-module and between-module degrees of every node (see Methods). Our results show that although modularization is insufficient to fully explain the greater-than-expected diameter in 8 of the 13 networks, it does explain a large fraction of the excess (Table S2).

### Implications

Despite the fact that real networks exhibit the small-world property and that shorter diameters may be beneficial to some networks, we show that all networks examined here, including biological networks, have diameters greater than their random expectations. We suggest that modularization may be a universal characteristic of real networks, due to the advantages it brings to network multi-functionality, robustness, and evolvability. As a consequence, the network diameter has to be sacrificed to accommodate modular structures. Because shorter diameters could provide higher functional efficiency, our result suggests a tradeoff between network efficiency and multi-functionality, robustness, and/or evolvability. Although there are many networks unstudied in this work, our analysis covers major types of networks and the results are likely to reflect a general pattern of real networks. This being said, it would be interesting to look for those rare networks whose diameters are shorter than the chance expectations and study what benefits offered by shorter diameters offset the advantages of modularity. In the case of biological networks such as the metabolic network or transcriptional regulatory network, it would be particularly interesting to examine the relationships among network diameter, modularity, and function.

## Methods

### Datasets

The sources of the 13 networks analyzed in this work are listed in Table S3.

### Modularity, diameter, and clustering coefficient

Modularity is defined according to Newman and Girvan [27]. Briefly, when the nodes of a network are separated into modules, one can compute 
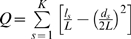
, where *K* is the number of modules, *L* is the total number of edges in the network, *l_s_* is the number of edges between nodes in module *s*, and *d_s_* is the total number of degrees of the nodes in module *s*. The highest *Q* value of all possible module separations is called the network modularity. In this work, we used the simulated annealing algorithm [22] to divide modules and calculate *Q*. Empirical and simulation studies showed that this algorithm has the best performance among all available algorithms because it provides the most accurate module separation and highest *Q* [*28*].

Diameter is defined as the average shortest path length over all pairs of nodes in the network and was calculated using the program “Topnet” [29]. For the yeast transcriptional regulatory network where the edges are naturally directed, we treated all edges as undirected for simplicity. Clustering coefficient of a node is the ratio of number of connections in the neighborhood of a node and the number of connections if the neighborhood was fully connected. Here neighborhood of node A means the nodes that are connected to A but does not include A itself. Clustering coefficient of a network is the mean clustering coefficient of all nodes and was calculated by “Topnet” [29].

### Randomly rewired networks

For a given network, we generated its randomly rewired networks by conserving the degree of every node, using the method previously described [30], [31]. Briefly, starting from a real network, the method randomly selects two edges from the network and swaps the connections under the condition that this exchange will not generate multiple edges between two nodes. For example, the algorithm changes an edge between nodes 1 and 2 and an edge between nodes 3 and 4 to an edge between 1 and 3 and an edge between 2 and 4. This process is repeated many times to produce a sufficiently randomized network. In this study, we generated 50 randomly rewired networks for each real network and computed the means and standard deviations of diameter and modularity of these 50 networks.

### Randomly rewired networks with conserved modules

To study if modularization is sufficient to explain the greater-than-expected diameter in real networks, we developed an algorithm to rewire a network randomly while preserving its original modules. First, we identify modules in a network using simulated annealing [22]. Second, we apply the random rewiring algorithm described above to each module. That is, we only rewire within-module edges by conserving the within-module degree of each node. Third, we randomly rewire inter-modular edges by conserving the between-module degree of each node. We generated 50 randomly rewired networks for each real network and computed the means and standard deviations of diameter and modularity of these 50 networks. The rewired network from these three steps will have a modularity that is either equal to or higher than that of the original network (Table S2). If modularization is sufficient to explain the high-than-expected diameter in real networks, the diameter of the rewired networks is expected to be close to that of the original network. However, the observed diameter is still greater than that of rewired networks in 8 of the 13 networks at 5% significance level (Table S2), suggesting that for these networks, modularization contributes partly, but not fully, to the excess of diameter over the random expectation. For 3 of the remaining 5 networks, the observed diameter is shorter than that of rewired networks, although the difference is not statistically significant. This phenomenon could be due to (i) stochastic error in estimating the expected diameter, (ii) imperfect design of the random rewiring with preserved modules, which produces networks with increased modularity, or (iii) presence of forces that reduce diameters under the constraint of a certain level of modularity.

### Computer simulation for investigating the relationship between modularity and diameter

Five sets of simulations were conducted. Within each set, all networks have the same numbers of nodes, edges, and modules, but different modularities. The networks were generated as previously described [15]. Briefly, the algorithm starts from a network of *m* fully connected modules, each having *M* nodes. Each pair of modules are connected by a single random edge. Then, the algorithm adds one node into a randomly selected module with *n = K+S* edges, where *K* is the number of within-module edges and *S* is the number of between-module edges. We used *n* = 31. These edges are attached to existing nodes via the preferential attachment model [7]. A total of *N* nodes are added. The degree distribution of the generated network was reported to approach the power law [15]. By adjusting parameters *K* and *S*, we can generate networks with desired modularity. The parameters used in each set of simulations are listed in Table S4.

After obtaining a simulated network, we conducted random network rewiring and computed *Z*-scores for diameter and modularity from 50 rewired networks (Fig. 1b). We also conducted random network rewiring by preserving modules and computed the mean diameter and mean modularity from 50 rewired networks. The relationship between the mean diameter and mean modularity (Fig. S3) is highly similar to that between diameter and modularity in the original simulated networks (Fig. 1a), indicating that the relationship we observed in Fig. 1a is not due to the specific means of network simulation, but reflects a general relationship between diameter and modularity.

### Generation of random power-law networks

To generate a power-law random network with a desired exponent, we adopted the Dorogovtsev–Mendes–Samukhin (DMS) method [18]. Briefly, a new node is added to the existing network and *m* edges are added simultaneously. The probability that node *i* attracts a link is 
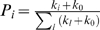
, with *−m<k*_0_ *<∞*. Here *k_i_* is the degree of node *i*, *l* is the set of all nodes in the network, *k_l_* is the degree of node *l*. This is a more general method than the standard BA model [7] because of the presence of the constant *k*_0_. For such attachment probability, one gets a power-law degree distribution with an exponent *γ = 3+k*_0_/*m*. Hence, as the initial attractiveness *k*_0_ grows from *−m* to *∞*, *γ* increases from 2 to *∞*. When *k*_0_ = 0, the model is equivalent to the standard BA model [7]. We generated networks with DMS model for the power and metabolic networks which have exponents of 2.75 and 2.40, respectively. The distributions of exponents in the simulated power-law networks for the power and metabolic networks are shown in Fig. S4 and Fig. S5, respectively. The means of exponents for the two generated network sets (50 networks in each set) are 2.73 and 2.38, respectively, close to the real ones.

## Supporting Information

Table S1 (0.01 MB PDF)

Table S2 (0.01 MB PDF)

Table S3 (0.01 MB PDF)

Table S4 (0.01 MB PDF)

Figure S1 Distributions of shortest path lengths in four representative networks. In each panel, closed bars are for the real network, whereas open bars are for a randomly rewired network. The networks presented are (a) the dolphin network, (b) the airline network, (c) the protein-protein interaction network, and (d) the electronic circuit network.
(0.08 MB PDF)

Figure S2 Correlation between network diameter and modularity in simulated networks when diameter and modularity are measured in absolute values. Each point represents a network and each line connects the networks of the same series. The number of modules is fixed at 2 for all series. The average degree is fixed at 49.7, 59.6, 62.25, 66.33, 99.56 and 99.6 for series A, B, C, D, E and F, respectively. Within each network series, the ratio (R) of the number of between-module edges to that of within-module edges changes from 20∶2 to 2∶20 to enhance modularity.
(0.12 MB PDF)

Figure S3 Correlation between network diameter and modularity in simulated networks. Each point represents a network and each line connects the networks of the same series. The number of modules is fixed at 4, 6, 8, 10 and 12 for series A, B, C, D, and E, respectively. Within each network series, the ratio (R) of the number of between-module edges to that of within-module edges changes from 30∶1 to 1∶30 so that modularity gradually increases. Here, the diameter and modularity values are averages from 50 randomly rewired networks (with preserved modules) of the original simulated networks. Error bars show one standard deviation.
(0.14 MB PDF)

Figure S4 The distribution of exponents in the 50 power networks simulated by the modified BA model. The real power network has an exponent of 2.75.
(0.12 MB PDF)

Figure S5 The distribution of exponents in the 50 metabolic networks generated by the modified BA model. The real metabolic network has an exponent of 2.40.
(0.12 MB PDF)
