# Supplementary material for: A Big World Inside Small-World Networks
Source: PLoS One. 2009 May 25;4(5):e5686. doi: 10.1371/journal.pone.0005686 (PMC2682646; doi:10.1371/journal.pone.0005686)
Supplement: Table S1 — (0.01 MB PDF) [file pone.0005686.s001.pdf]

Table S1. Clustering coefficients of 13 real networks.

Networks	# of nodes	Clustering coefficients: Observed	Expected	% difference	*Z* -score	*P* -value	Clustering coefficient and diameter: Correlation [1]	*P* -value
Characters in "Les Miserables"	77	0.37	0.33	10.7	3.42	0.0006	0.02	0.6226
Words in "David Copperfield"	112	0.09	0.09	1.6	0.15	0.8771	-0.09	0.0380
Dolphins	62	0.15	0.15	-2.4	-0.25	0.8026	0.23	$<10^{-4}$
Political blogs	1224	0.18	0.1584	13.6	3.64	0.0003	-0.06	0.3259
Co-authorship	7610	0.44	0.40	8.8	1.28	0.1996	0.03	0.5623
Football	115	0.20	0.19	8.6	6.15	$<10^{-4}$	0.53	$<10^{-4}$
Power	4941	0.05	0.05	6.8	0.64	0.5212	-0.04	0.3685
Airline	810	0.34	0.22	57.2	20.95	$<10^{-4}$	-0.42	$<10^{-4}$
Electronic circuits	512	0.03	0.03	12.8	0.79	0.4320	0.16	0.0004
Protein-protein interaction	1870	0.07	0.06	13.5	2.00	0.0455	-0.05	0.2746
Neural	297	0.15	0.11	41.8	18.92	$<10^{-4}$	-0.04	0.3410
Transcriptional regulatory	3459	0.05	0.05	8.8	2.44	0.0145	0.04	0.0048
Metabolic	563	0.05	0.04	15.4	0.96	0.3391	-0.02	0.6430

[1] Spearman's correlation between clustering coefficient and diameter among randomly rewired networks
