# Supplementary material for: A Big World Inside Small-World Networks
Source: PLoS One. 2009 May 25;4(5):e5686. doi: 10.1371/journal.pone.0005686 (PMC2682646; doi:10.1371/journal.pone.0005686)
Supplement: Table S2 — (0.01 MB PDF) [file pone.0005686.s002.pdf]

Table S2. The diameters and modularities of 13 real networks compared to the expected values in randomly rewired networks of conserved modules.

		Diameter						Modularity				
Networks	# of nodes	Observed	Expected	% difference	*Z* -score	*P*- value	% explainable[1]	Observed	Expected	% difference	*Z* -score	*P* -value
Miserables"	77	2.64	2.71	-2.5	-1.40	0.16	149	0.56	0.58	-3.45	-2.59	0.0096
Words in "David Copperfield"	112	2.54	2.54	-0.2	-0.20	0.84	107	0.31	0.36	-14.80	-4.87	$<10^{-4}$
Dolphins	62	3.36	3.07	9.3	4.53	$<10^{-4}$	56	0.53	0.56	-5.76	-2.77	0.0056
Political blogs	1224	2.74	2.71	1.0	4.45	$<10^{-4}$	81	0.43	0.45	-5.12	-21.21	$<10^{-4}$
Co-authorship	7610	7.03	5.74	22.4	97.40	$<10^{-4}$	20	0.81	0.81	0.48	0.08	0.9362
Football	115	2.51	2.44	2.8	7.46	$<10^{-4}$	76	0.60	0.61	-0.89	-2.73	0.0063
Power	4941	18.99	12.10	57.0	47.30	$<10^{-4}$	35	0.93	0.91	2.44	3.43	0.0006
Airline	810	3.06	3.11	-1.5	-1.90	0.06	110	0.31	0.39	-20.80	-3.52	0.0004
Electronic circuits	512	6.86	6.51	5.3	0.52	0.60	71	0.81	0.80	1.41	0.95	0.3421
Protein-protein interaction	1870	6.81	6.09	11.8	7.79	$<10^{-4}$	30	0.81	0.81	0.32	-0.39	0.6965
Neural	297	2.46	2.43	1.0	1.97	0.05	76	0.40	0.45	-11.29	-12.58	$<10^{-4}$
Transcriptional regulatory	3459	3.72	3.61	3.0	9.04	$<10^{-4}$	67	0.60	0.62	-2.71	-6.58	$<10^{-4}$
Metabolic	563	8.78	8.07	8.8	2.63	0.009	68	0.84	0.84	0.00	-0.35	0.7263

[1] (expected diameter of rewired networks with conserved modules - expected diameter of completely rewired networks)/(observed diameter-expected diameter of completely rewired networks) x 100.
% explainable is greater than 100 for three networks, possibly because of (i) stochastic error in estimating the expected diameter, (ii) imperfect design of the random rewiring with preserved modules, which produces networks with increased modularity, or (iii) presence of forces that reduce diameters under the constraint of a certain level of modularity.
