# Supplementary material for: A Big World Inside Small-World Networks
Source: PLoS One. 2009 May 25;4(5):e5686. doi: 10.1371/journal.pone.0005686 (PMC2682646; doi:10.1371/journal.pone.0005686)
Supplement: Table S3 — (0.01 MB PDF) [file pone.0005686.s003.pdf]

Table S3. Sources of the 13 networks analyzed in this work

Networks	Sources
Characters in "Les Miserables"	[1]
Words in "David Copperfield"	[2]
Dolphins	[3]
Political blogs	[4]
Co-authorship	[5]
Football	[6]
Power	[7]
Airline	http://www.transtats.bts.gov
Electronic circuits	http://www.weizmann.ac.il/mcb/UriAlon/
Protein-protein interaction	[8]
Neural	http://www.wormatlas.org/neurons.htm/neuronalconninfo.htm
Transcriptional regulatory	[9]
Metabolic	[10]

1. Knuth, D. E. The stanford graphbase: A Platform for Combinatorial Computing. *Addison-Wesley* (1993).
2. Newman, M. E. J. Finding community structure in networks using the eigenvectors of matrices. *Preprint physics/0605087* (2006).
3. D. Lusseau, K. S., O. J. Boisseau, P. Haase, E. Slooten, and & Dawson, S. M. The bottlenose dolphin community of doubtful sound features a large proportion of long-lasting associations. *Behav Ecol Sociobiol* 54, 396-405 (2003).
4. Adamic, L. A. & Natalie, G. The political blogosphere and the 2004 us election. *Proceedings of the WWW-2005 Workshop on the Weblogging Ecosystem* (2005).
5. Newman, M. E. J. The structure of scientific collaboration networks. *Proc. Natl Acad. Sci.U S A* 98, 404-409 (2001).
6. Girvan, M. & Newman, M. E. J. Community structure in social and biological networks. *Proc. Natl Acad. Sci.U S A* 99, 7821-7826 (2002).
7. Watts, D. J. & Strogatz, S. H. Collective dynamics of 'small-world' networks. *Nature* 393, 440-442 (1998).
8. Jeong, H., Mason, S., Barabási, A.-L. & Oltvai, Z. N. Centrality and lethality of protein networks. *Nature* 411, 41-42 (2001).
9. Luscombe, N. M. et al. Genomic analysis of regulatory network dynamics reveals large topological changes. *Nature* 431, 308-312 (2004).
10. Ma, H. & Zeng, A. P. Reconstruction of metabolic networks from genome data and analysis of their global structure for various organisms. *Bioinformatics* 19, 270-277 (2003).
