# Supplementary material for: A Big World Inside Small-World Networks
Source: PLoS One. 2009 May 25;4(5):e5686. doi: 10.1371/journal.pone.0005686 (PMC2682646; doi:10.1371/journal.pone.0005686)
Supplement: Table S4 — (0.01 MB PDF) [file pone.0005686.s004.pdf]

Table S4. Parameters used in each set of simulations used for generating networks for Fig. 1

Simulations	*m*	*M*	*N*	*S*	*K*
Series A	4	30	130	From 30 to 1	From 1 to 30
Series B	6	30	130	From 30 to 1	From 1 to 30
Series C	8	30	130	From 30 to 1	From 1 to 30
Series D	10	30	130	From 30 to 1	From 1 to 30
Series E	12	30	130	From 30 to 1	From 1 to 30
