# Supplementary figures and images for: A Big World Inside Small-World Networks
Source: PLoS One. 2009 May 25;4(5):e5686. doi: 10.1371/journal.pone.0005686 (PMC2682646; doi:10.1371/journal.pone.0005686)

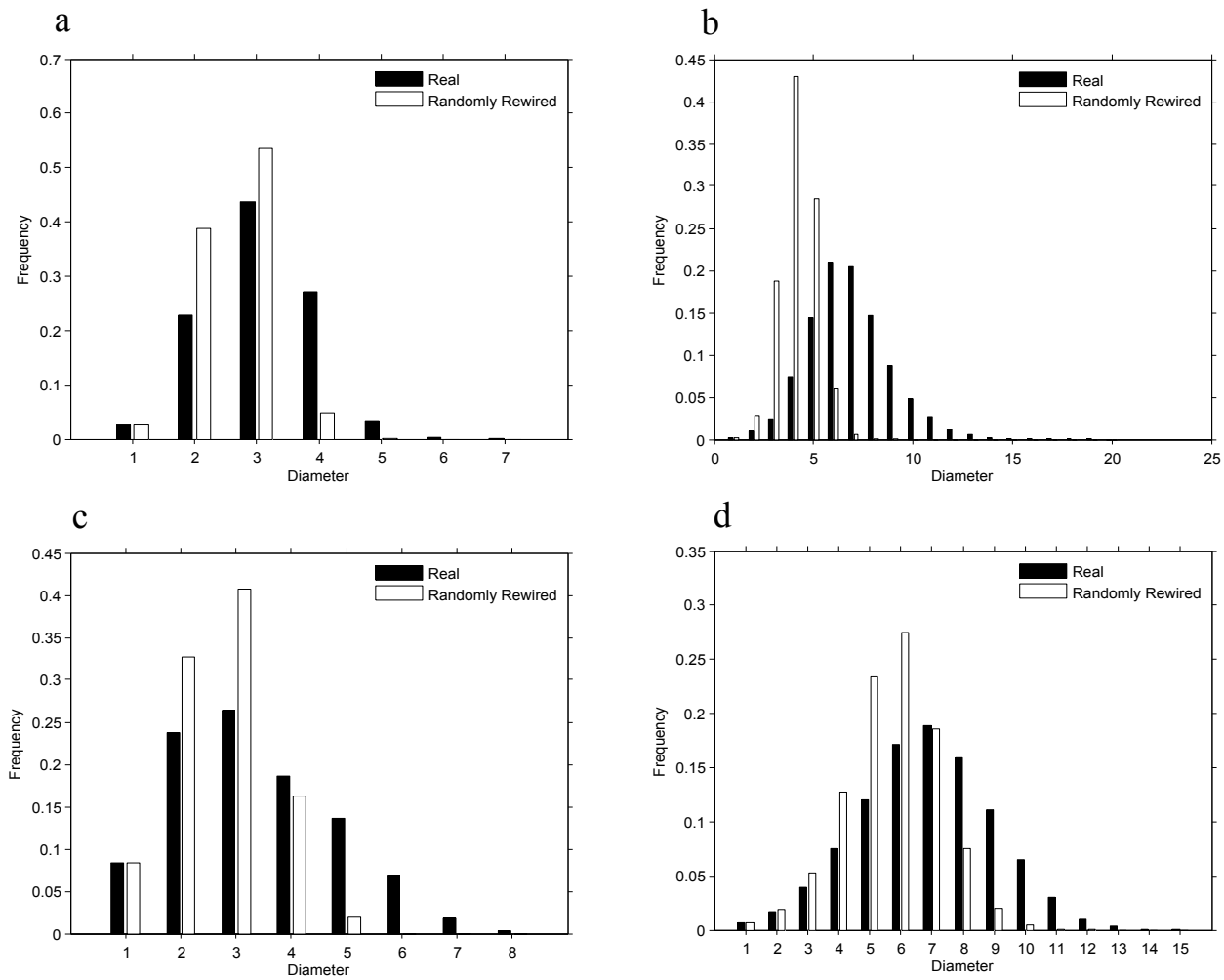

Figure S1

Supplement: Figure S1 — Distributions of shortest path lengths in four representative networks. In each panel, closed bars are for the real network, whereas open bars are for a randomly rewired network. The networks presented are (a) the dolphin network, (b) the airline network, (c) the protein-protein interaction network, and (d) the electronic circuit network. (0.08 MB PDF) [file pone.0005686.s005.pdf]

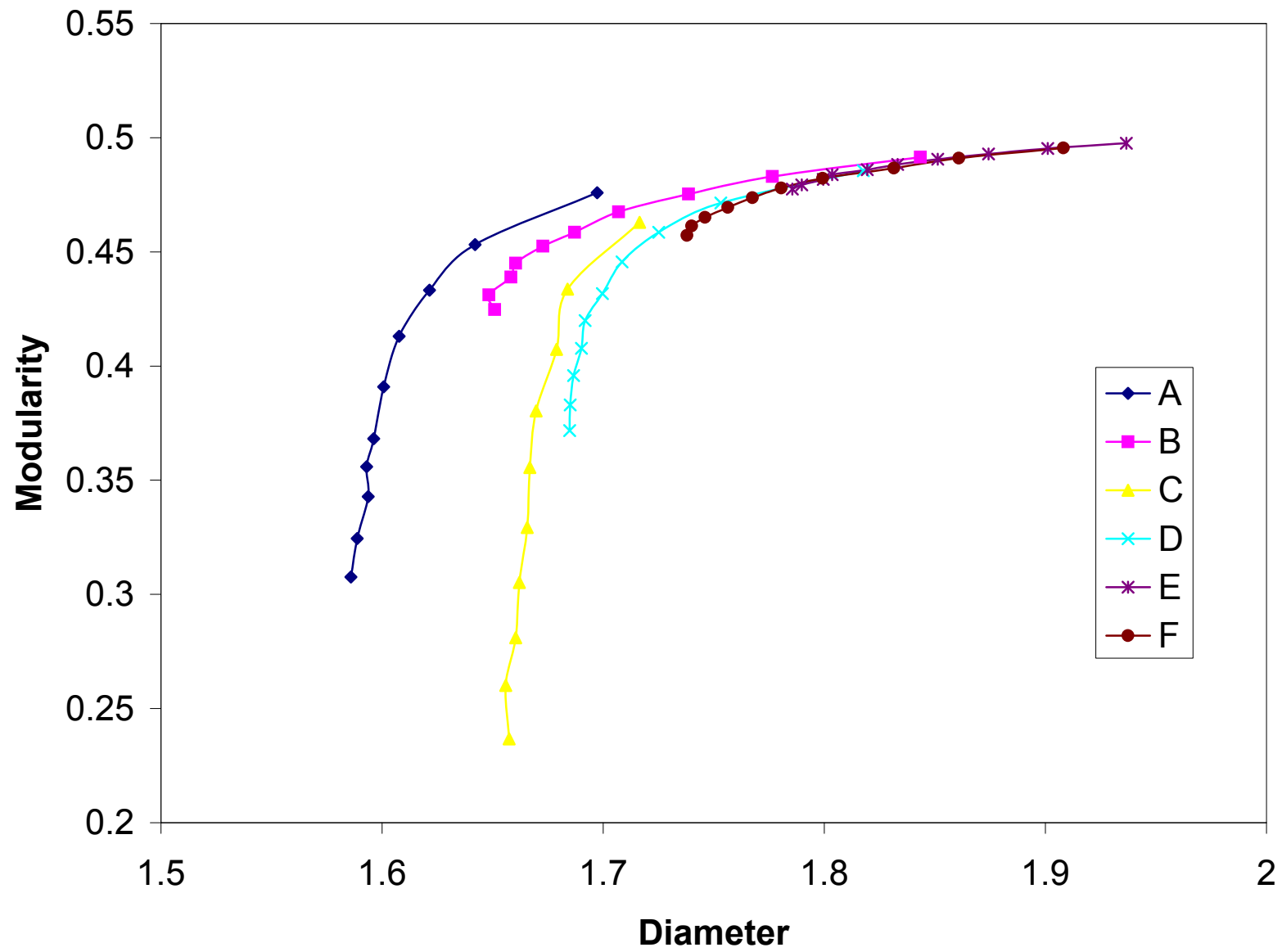

Figure S2

Supplement: Figure S2 — Correlation between network diameter and modularity in simulated networks when diameter and modularity are measured in absolute values. Each point represents a network and each line connects the networks of the same series. The number of modules is fixed at 2 for all series. The average degree is fixed at 49.7, 59.6, 62.25, 66.33, 99.56 and 99.6 for series A, B, C, D, E and F, respectively. Within each network series, the ratio (R) of the number of between-module edges to that of within-module edges changes from 20∶2 to 2∶20 to enhance modularity. (0.12 MB PDF) [file pone.0005686.s006.pdf]

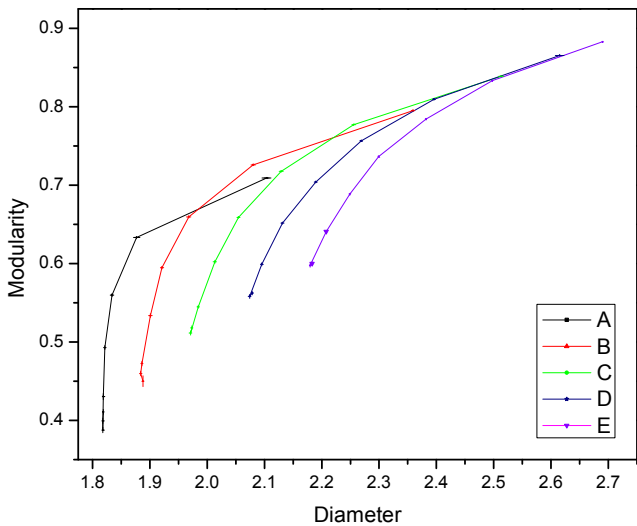

Figure S3

Supplement: Figure S3 — Correlation between network diameter and modularity in simulated networks. Each point represents a network and each line connects the networks of the same series. The number of modules is fixed at 4, 6, 8, 10 and 12 for series A, B, C, D, and E, respectively. Within each network series, the ratio (R) of the number of between-module edges to that of within-module edges changes from 30∶1 to 1∶30 so that modularity gradually increases. Here, the diameter and modularity values are averages from 50 randomly rewired networks (with preserved modules) of the original simulated networks. Error bars show one standard deviation. (0.14 MB PDF) [file pone.0005686.s007.pdf]

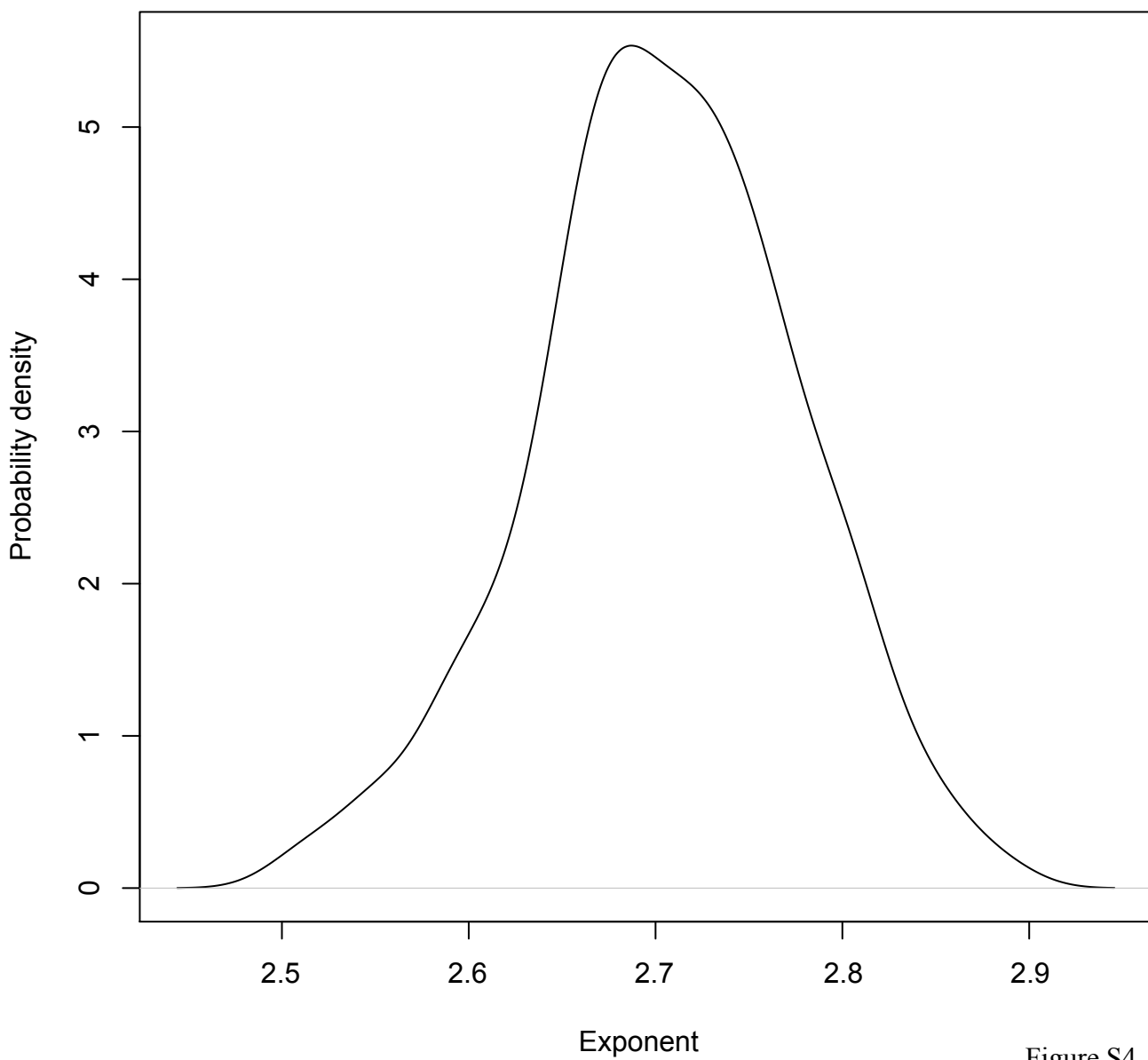

Figure S4

Supplement: Figure S4 — The distribution of exponents in the 50 power networks simulated by the modified BA model. The real power network has an exponent of 2.75. (0.12 MB PDF) [file pone.0005686.s008.pdf]

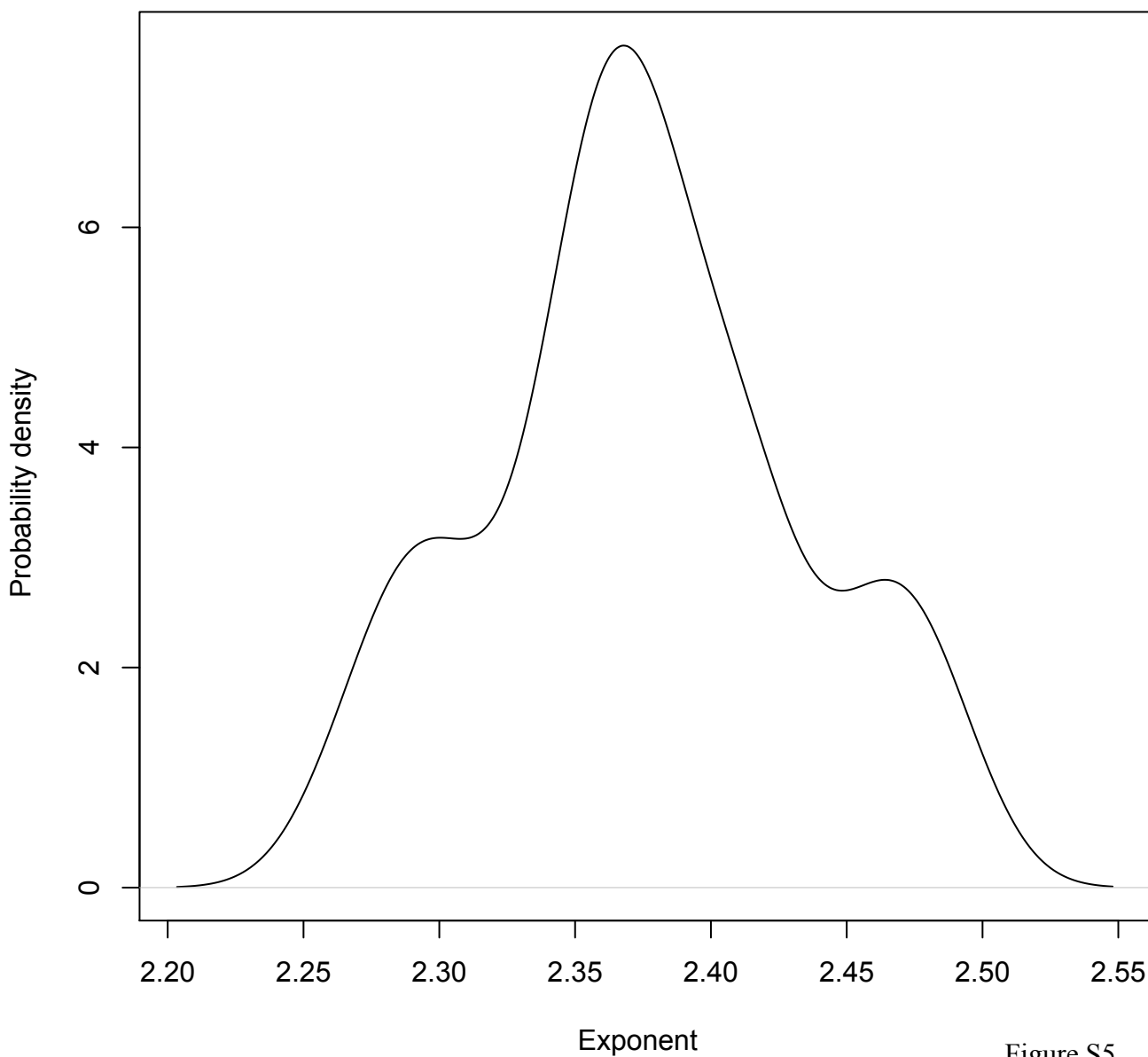

Figure S5

Supplement: Figure S5 — The distribution of exponents in the 50 metabolic networks generated by the modified BA model. The real metabolic network has an exponent of 2.40. (0.12 MB PDF) [file pone.0005686.s009.pdf]
